# Implementing a community specialist team to support the delivery of integrated diabetes care: experiences in Ireland during the COVID-19 pandemic

**DOI:** 10.12688/hrbopenres.13635.1

**Published:** 2023-01-05

**Authors:** Fiona Riordan, Lauren O'Mahony, Cormac Sheehan, Katie Murphy, Maire O'Donnell, Lorna Hurley, Sean Dinneen, Sheena M. McHugh

**Affiliations:** 1 School of Public Health, University College Cork, Cork, Ireland; 2 Department of General Practice, University College Cork, Cork, Ireland; 3 School of Medicine, National University of Ireland, Galway, Galway, Ireland; 4 Integrated Care Programme for Chronic Disease, Health Service Executive, Ireland, Ireland; 5 Centre for Diabetes, Endocrinology and Metabolism, University Hospital Galway, Galway, Ireland

**Keywords:** implementation, diabetes, integrated care

## Abstract

**Background: **While models of integrated care for people with chronic conditions have demonstrated promising results, there are still knowledge gaps about *how* these models are implemented in different contexts and which strategies may best support implementation. We aimed to evaluate the implementation of a multidisciplinary diabetes Community Specialist Team (CST) to support delivery of integrated type 2 diabetes care during COVID-19 in two health networks.

**Methods: **A mixed methods approach was used. Quantitative data included administrative data on CST activity and caseload, and questionnaires with GPs, practice nurses (PN) and people with type 2 diabetes. Qualitative data were collected using semi-structured interviews and focus groups about the service from CST members, GPs, PNs and people with type 2 diabetes. We used the Consolidated Framework for Implementation Research framework to explain what influences implementation and to integrate different stakeholder perspectives.

**Results: **Over a 6-month period (Dec 2020-May 2021), 516 patients were seen by podiatrists, 435 by dieticians, and 545 by CNS. Of patients who had their first CST appointment within the previous 6 months (n=29), 69% (n=20) waited less than 4 weeks to see the HCP. During initial implementation, CST members used virtual meetings to build ‘ *rapport’* with general practice staff, supporting ‘ *upskilling’* and referrals to the CST. Leadership from the local project team and change manager provided guidance on how to work as a team and ‘ *iron out’* issues. Where available, shared space enhanced networking between CST members and facilitated joint appointments. Lack of administrative support for the CST impacted on clinical time.

**Conclusions: **This study illustrates how the CST benefited from shared space, enhanced networking, and leadership. When developing strategies to support implementation of integrated care, the need for administrative support, the practicalities of co-location to facilitate joint appointments, and relative advantages of different delivery models should be considered.

## Introduction

International health systems are increasingly focused on implementing innovative and more efficient models of integrated care for chronic disease which fit with the local context ^1^, address service fragmentation, and improve patient experience ^2, 3^. The World Health Organisation defines integrated care as “the management and delivery of health services such that people receive a continuum of health promotion, health protection and disease prevention services, as well as diagnosis, treatment, long-term care, rehabilitation, and palliative care services through the different levels and sites of care within the health system and according to their needs” ^4^. Integrated care focuses on care coordination around the patient, either through vertical integration between acute and community or social care services, or horizontal integration across services at a similar level ^5, 6^.

While some evaluations of integrated care show promising results in terms of patient outcomes (preventable hospitalisations, bed-days, clinical outcomes) ^5, 7, 8^, findings have been inconsistent ^9– 12^. Proposed reasons for the inconsistencies are that integrated care models are complex and difficult to implement in real world settings. Furthermore, evaluations tend to focus on determining ‘if’ integrated care works based on outcomes such as reducing hospital admissions that can be affected by a range of factors and may not be directly attributable to the integrated care model ^12^. Despite the proliferation of integrated care programmes and interventions, there are still gaps in our knowledge of *how* these models of care are implemented in different contexts ^10, 12, 13^ and which strategies may work best to support implementation ^14^. For example, while multidisciplinary teams may be technically fully implemented to support integrated care, nuanced challenges in terms of *how* the team works could impact on implementation and may be missed by evaluators ^12^. Authors in the field have called for implementation models, theories, and frameworks ^14^ to be used to describe and explain what influences implementation and whether factors are operating at the system, organisation or individual professional or patient level ^15^. Doing so would inform future strategies and adaptations to improve the fit of these strategies with the service context and allow for shared learning across different settings.

Type 2 diabetes, with its increasing prevalence ^16, 17^ and significant burden on the individual and the wider health system ^1, 18, 19^ is often used as an exemplar condition when developing, testing, and evaluating new models of integrated care. Models of integrated diabetes management often involve realignment of services in different settings according to risk or need, for example, management of people with uncomplicated diabetes in primary care, with management of complicated diabetes between primary and secondary or solely in secondary care ^4, 20^, Strategies to support implementation of integrated diabetes care models typically include workforce changes and new ways of working ^21^, including the creation of new clinical staff/teams (e.g., co-location of specialities ^20, 22, 23^, introduction of new staff/roles, often specialist nurses ^24, 25^), revision of professional roles ^26^, ongoing training (e.g., upskilling of general practitioners/practice nurses ^5, 23, 27, 28^), or learning collaboratives (e.g., joint meetings to support greater multidisciplinary team working ^24, 29^).

Few studies have examined the process of implementing community specialist teams to support delivery of integrated diabetes care ^20, 22, 23^. In Ireland, as part of the Sláintecare reform programme, co-located specialist services (Clinical Nurse Specialist (CNS), Dietitian, and Podiatrist), supported by a change manager, were piloted in two health networks. This study investigated the introduction of a CST during the COVID-19 pandemic when health systems, including those in Ireland, were dealing with disruption to routine care and a shift to remote delivery ^30^. Using tools and perspectives from implementation science, we evaluated the introduction of the CST to understand barriers and facilitators to implementation of the strategy as well as adaptations due to COVID-19.

## Methods

### Design

A mixed methods approach was used, involving parallel quantitative and qualitative data collection among healthcare professionals (the CST and general practice staff) and people with diabetes in two community health network areas (Network 7 and Network 9). We integrated all data at the level of interpretation and reporting ^31^. The study is reported in accordance with the Standards for Reporting Implementation Studies (StaRI) ^32^.

### Setting

In Ireland, there is no universal access to primary care. People who hold a General Medical Services (GMS) card (32.4%) or GP visit card (GPVC) (10.4%) ^33^ are eligible for free GP visits, hospital care and medications (except for a prescription levy), and eligibility is largely based on income ^34^. Non-GMS or ‘private’ patients either pay to receive diabetes care from their GP or attend secondary care with no fee at the point of access. People with chronic diseases can apply to the Long Term Illness Scheme which is not based on income; under this scheme people can access some drugs and medicines for free ^35^. Services provided by the CST were accessible by both GMS and non-GMS patients. The Chronic Disease Management Programme reimburses general practices for structured care for people diagnosed with different chronic conditions, including type 2 diabetes. People are eligible for the programme based on holding a GMS card ^36, 37^. 

In line with the introduction of the Enhanced Community Care programme, health services are organised according to 96 Community Health Networks (CHN), which deliver primary healthcare services for a population of 50,000. The CST initiative was piloted in two health networks in the West (Network 7) and South (Network 9) of Ireland. A designated GP Lead represents GPs at a Network level; they have one day each week dedicated to work in the network, representing GP views at meetings, liaising with their GP colleagues, and co-designing services.

The two networks in this pilot, in the West (Network 7) and South (Network 9) of Ireland respectively, represent different levels of diabetes care delivery in primary and secondary care and cover different areas of socio-economic deprivation; in Network 9 the level of disadvantage is higher (4% below the national average) than in Network 7 (1% above the national average). Within Network 9 there is a local general practice led initiative whose aim is to provide a forum for GPs and practice nurses to ensure best practice in their management of diabetes in general practice through peer support, education and audit ^38^.

***Study context: COVID-19 restrictions***

At the time of the study, in December 2020 – July 2021, two national lockdowns took place due to the COVID-19 pandemic (October 2020-early December 2020, and January 2021 to April 2021). As part of these restrictions, those over 70 and the medically vulnerable were advised to limit contacts outside the home and people were reluctant to attend their GP or outpatient appointments ^39^. Where possible healthcare services were offered virtually, with a focus on priority and urgent care ^40^. 

### Intervention

CST were recruited in two networks to support delivery of a model of integrated care for type 2 diabetes ^41^, whereby patients with uncomplicated type 2 diabetes are managed in primary care, and patients with complicated type 2 diabetes are managed between primary and secondary care. Uncomplicated type 2 diabetes is defined as people with the condition who are not on insulin, their diabetes is managed by lifestyle modifications only or they are on 2 glucose lowering agents with IFCC/HBA1c (< 58mmol/l or <7.5%). They also are at low risk of complications (for example, renal function, eye disease, diabetic foot). Complicated type 2 diabetes is defined as people who have a need for insulin, IFCC/HBA1c (> 58mmol/l or 7.5%) or have or are at high risk of complications (e.g., active, or high-risk foot, renal failure). Full definitions are available in the guidance ^41^. General practitioners (GPs) have a key role in managing uncomplicated type 2 (recommended visits three times a year in GP practice) and sharing management of complicated type 2 (seen up to twice a year in GP practice) with support from specialists in secondary care and the community. As part of the model the CST deliver one-to-one patient education and patient appointments in the community.

### Implementation strategy

Implementation of the model of care was supported by the introduction of several strategies, with the diabetes CST as the main implementation strategy. The following strategies, as mapped to the Expert Recommendations on Implementing Change (ERIC) compilation ^42^, were introduced in the two networks ( Table 1).

**Table 1.** Strategies to support the implementation of the model of care mapped to the ERIC compilation.

ERIC	Description
**Creation of new clinical ** **teams**	The CST were introduced and advertised as a team to primary care practitioners (GPs, practice nurses, public health nurses) in the networks. A full team (CNS, dietitian, and podiatrist) was recruited in Network 7, but there were difficulties recruiting a CNS in Network 9 during the project timeline. Therefore, two pre-existing CNSs who were not formally part of the pilot, delivering diabetes clinics in general practice in the network, and covered a much larger geography, provided some nursing input to the Network 9 team.
**Change to physical structure** **and equipment**	A key aspect of the strategy was co-location (clinicians in Network 9 were based in the same office building whereas those in Network 7 shared office space).
**Create a learning** **collaborative**	Case discussions were facilitated through by podiatrists and CNS in both networks (20% Whole Time Equivalent (WTE) spent in hospital) and the establishment of fortnightly MDT meetings with Consultant Endocrinologists (Network 7).
**Conduct educational** **meetings, ongoing training,** **and shadow other experts**	Primary care health professional education, including training workshops, shadowing opportunities, and educational presentations during practice visits, were delivered by all members of the CST. These meetings and training opportunities were intended to support management of referrals to and from the CST in accordance with GP referral /eligibility criteria agreed for each clinician [available as extended data ^43^] along with the process for managing new referrals.
**Develop and organize quality** **monitoring systems**	Routine monitoring of the service was conducted; CST members collected data on their activity and caseload throughout the pilot project.
**Facilitation**	Introduction of a change manager to support implementation at each site.

### Data collection

Quantitative and qualitative date were collected from multiple stakeholder groups ( Table 2). 

**Table 2.** Overview of data collection.

Source	Method	Total participants
**CST**	**Activity data** collected over a 6-month period (December 2020 to May 2021) **Caseload data** collected over two 3-month periods: Dec. to Feb. and March to May	All 3 members of the CST collected data in Network 7. Complete data were collected by the dietician and podiatrist in Network 9. Partial activity data collected by the 2 CNS in Network 9; incomplete data on episodes, referrals, and education, and no data on caseload.
**CST and change** **manager**	**Semi-structured interviews** conducted in July 2021 (online or phone)	7 HCPs (2 podiatrists, 2 dietitians, 3 CNS) 1 change manager.
**General** **practice staff**	**Semi-structured interviews** with GPs, and **focus** **groups** with practice nurses (online)	6 GPs 2 focus groups conducted with 9 practice nurses.
	**Survey** between December 2020- January 2021, self-completed by practices or administered by a CST member. Closed and open questions on current diabetes care delivery at their practice.	52% (n=15/29) practices
**People with** **Type 2 diabetes**	**Survey** administered to people who had attended the CST during the first 2 weeks in May 2021	49% (n=41/85)
	**Semi-structured interviews** with a purposive sample of patients who indicated on the questionnaire that they were willing to take part in a short follow-up telephone interview	31/41 (76%) consented to be contacted by a researcher to take part in a telephone interview. 9 participants were interviewed.

***Reflexivity***

Interviews and focus groups were conducted by FR, CS and MOD. All are non-clinical postdoctoral health services researchers based in academic departments within the University College Cork (FR, CS) and University of Galway (MOD). All had experience interviewing health care professionals and patients. Participants knew the interviewers as independent researchers conducting the evaluation on behalf of the team responsible for implementing the new model of care and clinical specialist team.

***Community Specialist Teams***

Each member of the CST collected data on their activity between December 2020 and May 2021 and active quarterly caseload during two 3-month periods: Dec. to Feb and March to May. These data included the numbers of new and return patients, education sessions delivered and referrals (number, type) from their service, number of appointments (and whether face to face or by telephone), number of education sessions delivered, number and type of referrals from their service. Caseload data included patient demographics (age, sex, diabetes type, GMS status), source of referrals, waiting list and the number of GP practices engaging with their service.

 Semi-structured (online or phone) individual interviews were conducted by FR in July 2021 with members of the CST and CNS already working in the network (Network 9), and the project change manager. LH initially highlighted the study with members of the CST who then followed up with FR if they were willing to participate. These interviews aimed to elicit their views on the acceptability and practicality of implementing the integrated care service, along with barriers and facilitators of implementation.

***General practice***

General practices in both networks, Network 7 (n=11) and Network 9 (n=18), were asked to complete a survey on diabetes care delivery at their practice. The survey was issued between December - January 2021 and included closed and open questions on the practice profile (i.e., practice size, staff, type 2 caseload, GMS, and private patients), delivery of structured care (recall and review)) access to specialist services and allied diabetes services and continuing professional development).

In July-August 2021, CS conducted semi-structured online interviews with GPs and focus groups with practice nurses. The aim was to explore their experience of delivering diabetes care and linking in with the CST and views on the CST service. The number of practice nurses and GPs sampled for the study was decided based on reaching data saturation, but also pragmatically based on GP availability given the demands placed by Covid-19 on practices at the time.

***People with diabetes***

A mixed methods approach using postal questionnaires and semi-structured interviews by phone was used to elicit patient experiences of the CST service. A sequential dependent mixed method design was used for this component of the study, where findings from the survey data informed the number and purposive selection of who to approach for the follow-up qualitative interviews. The questionnaire asked for the name and speciality of the CST member the person had attended, measured different dimensions of integrated care informed by existing US and UK survey questionnaires ^44, 45^ (accessibility of the community diabetes service, communication with the patient and between health care professionals, access to information and person-centredness of the consultation). The questionnaire also included the 5-item CARE person-centred process measure ^46^ which measures person-centredness and empathy during a one-on-one consultation between a health care professional and a patient. Three open-ended questions asked respondents to comment on positive aspects about the consultation, aspects that could be improved and any general overall feedback on their diabetes care. The questionnaire was pilot tested. Questionnaires were posted out to people with type 2 diabetes who had attended a member of the CST during the first 2 weeks of May 2021.

Respondents were purposively selected (based on age, gender, diabetes duration, source of referral, number of HCPs attended and number of visits) from those who had indicated on the questionnaire they were willing to take part in a short follow-up semi-structured interview to explore their experience of attending the CST in greater detail.

For all interviews and focus groups, only participants and researchers were present.

The topic guides were developed with the input of all authors through discussion. The patient topic guide was informed by the questions asked in the questionnaire then tailored based on respondent’s responses to the questionnaire. The general practice staff interview guide was based on emerging data from the questionnaires and was amended to a small degree as the interviews took place and led to unstructured questions during the focus groups.

Topic guides used for CST interviews were pilot tested with members of the research team with a clinical background. Repeat interviews were not carried out. Researchers made field notes after each interview or focus group. Copies of all topic guides and questionnaires are publicly available ^43^.

### Recruitment

Surveys (practice and patient) were posted by the project change manager to general practices, and to eligible people with type 2 diabetes who had received a flyer from a member of the CST advertising the survey. Members of the CST were recruited for interview through the change manager and followed up by FR by email to arrange an interview. General practice staff at each site were contacted by the CST to advertise the evaluation, and those interested in taking part in an interview were contacted the research team (CS) directly, initially by e-mail. The researcher (MOD) contacted each person with diabetes who agreed on the questionnaire to be contacted for follow-up interview, in the first instance contacting them by text message to agree a time to speak to them over the phone.

Before completing interviews and surveys interview, all participants were invited to read a participant information sheet and complete a consent form. 

### Data analysis

***Activity data***

CST activity data were analysed descriptively in Excel (Microsoft 365, Excel Version 2208) by FR. Patients seen and patient episodes were reported as total and mean (sd) per month. Referrals, education sessions delivered and the number of professionals and patients attending the session were reported as total and median (range i.e., min, max) per month. Patient caseload data were reported as frequencies and percentages or mean (sd).

***Survey data***

Survey (practice and patient) data were entered into Excel before importing into SPSS version 27 for further cleaning, coding, and statistical analysis by LOM. Descriptive statistics were used to summarize and analyse characteristics. Data were reported as median (range) and frequencies and percentages.

GP practice data were analysed to generate descriptive statistics for the practices in each network separately and overall. Median caseload per GP was calculated by dividing the total practice caseload by the number of Whole Time Equivalent (WTE) GPs, assuming balanced caseload across all GPs. As some respondents did not provide figures for the total practice patient population, the total was calculated by summing the number of GMS patients and non-GMS (private) patients. Similarly, total type 2 diabetes patient caseload was calculated from T2D GMS and T2D non-GMS (private). All results are reported as the % of practices who responded to that question. Open-ended survey responses were analysed thematically, for example, where there were consistencies across several of the responses.

***Interview and focus group data***

Interviews and focus groups were audio-recorded and transcribed verbatim, and data managed using NVivo software (Version 12). Interviews with the CST were analysed using the Framework Method. Specifically, they were analysed deductively by FR and LOM using the Consolidated Framework for Implementation Research (CFIR) framework, utilizing the CFIR codebook adapted for the project and guided by the rapid analysis approach used by Keith *et al.* ^47^. CFIR is a conceptual framework commonly used in implementation research to systematically identify and group factors which influence the implementation of health service interventions was used to guide the analysis ^47^. Before coding to CFIR each transcript was coded to 6 core components of the CST:

1. Working as a team, advertised to primary care practitioners (GPs, practice nurses, public health nurses)
2. Managing referrals to and from the CST
3. Conducting health care professional education
4. Conducting patient education
5. Conducting patient appointments
6. Routine monitoring of the service (routine monitoring is an expected component of the CST intervention; however additional data were collected to fulfil reporting and evaluation requirements for the project).

**Figure 1.**
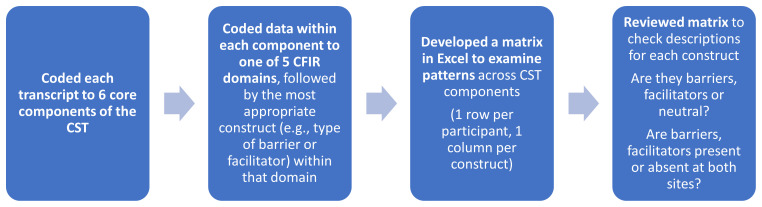
Overview of the coding approach using CFIR.

Coding was done independently initially by two researchers (FR and LOM) and then compared to ensure consistency in the use of the CFIR codebook. Details of the coding process are provided in Figure 1. Broad themes were developed based on contextualizing the most common constructs (barriers/facilitators) and considering *how* they influenced implementation. Throughout the analysis, the research team met regularly so that additional queries about coding were discussed to resolve any uncertainties.

Qualitative data collected from GPs and practice nurses and patients were analysed separately using reflexive thematic analysis following guidance from Braun and Clarke ^48^.

Member checking was not conducted.

### Integration

We integrated all types of data ^49^. As the CST represented the main component of the implementation strategy, themes developed from the CST interviews using CFIR provided the overarching organising structure. We led with the qualitative data collected from members of the CST and supplemented the main themes (those common across networks) with data from other stakeholder perspectives; themes from the analysis of GP, practice nurses and patients were mapped to the relevant CFIR construct to build on, or provide a different perspective to, the findings from the CST.

## Results

All members of the CST and the change manager took part in interviews, including the dietician, podiatrist in Network 7 and 9, one CNS in Network 7 and two CNS in Network 9. Interviews were conducted with six GPs and two focus groups conducted with nine practice nurses. On average, CST interviews were one hour in duration, GP interviews lasted 20-30 minutes, and focus groups took 90 minutes.

Overall, 52% (n=15/29) practices and 49% (n=41/85) patients completed the questionnaires. Of the 41 questionnaires completed, 31 (76%) survey respondents consented to a short follow-up interview and provided their name and telephone number for contact purposes. Nine were interviewed. Based on the overall positive experiences reported in the initial survey questionnaire data and the subsequent positive experiences reported in these initial 9 interviews, it was decided not to carry out any further interviews. Interview duration was on average was 14 minutes.

For general practice staff, data saturation was judged to have been achieved by the second focus group, as no new information was forthcoming. Data saturation was decided through discussion with the second facilitator after the focus groups.

All three members of the CST collected data in Network 7. Complete data were collected by the dietician and podiatrist in Network 9. Partial activity data collected by the 2 CNS in Network 9; incomplete data on episodes, referrals, and education, and no data on caseload.

Details on the characteristics of practices and patients surveyed and professionals and patients who took part in focus groups and interviews are available in Table 3 and Table 4.

**Table 3.** Characteristics of practices surveyed (n=15) and GP and PN interviewees and focus group participants (n=12).

Practices		Overall (N=15)	Network 9 (N=10)	Network 7 (N=5)
		Median (range)	Median (range)	Median (range)
**N staff (WTE)**	GPs	3.0 (1.0-9.0)	2.5 (1.0-9.0)	3.5 (2.5-6.0)
	GP Registrar ^α^	1.0 (1.0-1.0)	1.0 (1.0-1.0)	1.0 (1.0-1.0)
	Practice Nurse ^§^	1.3 (0.5-5.0)	1.3 (0.5-5.0)	1.5 (1.0-4.0)
	Practice Manager ^¶^	1.0 (0.5-2.0)	1.0 (0.5-2.0)	1.0 (1.0-1.0)
	Other admin. staff	3.0 (1.0-12.0)	2.5 (1.0-12.0)	3.0 (2.5-4.0)
**Total population**	Overall ^β^	4500 (700-16000)	2750 (700-11288)	7557 (4000-16000)
	GMS/GPVC ^†^	1700 (440-4348)	1000 (440-3082)	2130 (1700-4348)
	Non-GMS (Private) ^§^	1800 (100-11652)	1500 (100-8206)	5777 (1600-11652)
**Diabetes caseload**	T2D Overall ^§^	147 (50-566)	174 (50-566)	131 (66-230)
	GMS/GPV ^¥^	104 (13-511)	100 (13-511)	107 (44-118)
	Private ^µ^	24 (5-70)	24 (5-70)	22 (11-26)
	T1D ^β^	18 (5-48)	18 (5-48)	17 (7-20)
	Other e.g., MODY ^‖^	0 (0-1)	.5 (0-1)	0 (0-0)
Interview/focus group participants		N		
**Profession**	GP	3		
	Practice nurse	9		
**Gender**	Male	1		
	Female	11		
**Network**	7	7		
	9	5		
**Location**	City	3		
	Town	9		

**Abbreviations:** SD, Standard Deviation; MODY, Maturity Onset Diabetes Of the Young; N/A, Not Available; GMS/GPVC, General Medical Scheme/General Practice Visit Card; T2D, Type 2 Diabetes; T1D, Type 1 Diabetes αOverall, n=3, Network 9, n=2, Network 7, n=1 ¶Overall, n=9, Network 9, n=6, Network 7, n=3 βOverall, n= 14, Network 9, n=10, Network 7, n=4. †Overall, n=13; Network 9, n= 9; Network 7, n=4 §Overall, n=12; Network 9, n=8; Network 7, n=4 ¥Overall, n=12; Network 9, n=9; Network 7, n=3 µOverall, n=10; Network 9, n=7; Network 7, n=3 ‖Overall, n=3; Network 9, n=2; Network 7, n=1

**Table 4.** Characteristics of respondents to the patient survey (n=41) and interviewees (n=9).

		Survey respondents		Interviewees
		N	%	N
**Gender**	Male	25	61	4
	Female	16	39	5
**Age**	< 40 years	1	2	-
	41-55 years	6	15	-
	56-65 years	12	29	-
	66-70 years	7	17	-
	>70 years	15	37	-
**Diabetes diagnosis**	Less than 12 months ago	10	24	-
	1-5 years ago	11	27	-
	More than 5 years ago	20	49	-
**Diabetes diagnosis**	> 10 years ago			4
	<10 years ago			5
**Referred by**	General practice			6
	Secondary care			3
**CST appointments**	More than one			7
	One appointment			2

**Abbreviations**: CST, Clinical Specialist Team

We present the findings in three sections: an overview of implementation status, barriers and facilitators to implementation, and adaptations during the pandemic. A more detailed overview of the factors influencing implementation at each site, including the impact of COVID-19, are outlined in extended data ^43^. 

### Implementation status

***Patients seen***

Over a 6-month evaluation period (December 2020-May 2021), 516 patients with type 2 diabetes were seen by podiatrists, 435 by dieticians, and 545 by CNSs in the two networks. A greater proportion of returning patients (compared to new patients) were seen across both networks: Network 7 (n= 485, 62%), and Network 9 (n=766, 75%). The number of patients seen by two or more members of the CST (i.e., shared patients) was not recorded.

***Access to the CST***

Where this information was collected (n=4 clinicians), the number of people with diabetes on waiting lists for their service increased during the evaluation period. GPs and practice nurses valued the accessibility of the CST as a locally delivered service with perceived shorter waiting times compared to outpatient services. The accessibility of the service was also emphasized by patients. Of those who reported having their first appointment within the previous 6 months (n=29), 69% (n=20) reported a waiting time of less than 4 weeks to see the HCP. Of those who had attended a face-to-face consultation (n=33), 88% (n=29) reported waiting less than 15 minutes to see the HCP on the day of their appointment and 74% (n=25) reported having to travel less than 5 miles to attend their appointment.

*“I was instantly called [into my appointment], I was never more than 5 minutes …to be called for my appointment….and I have been 2 or 3 hours (waiting) in (outpatient clinic) and trying to park the car was unreal…” (Patient#4, Attended Podiatrist).*

## Barriers and enablers to implementation of the CST

### Inner setting

***Available resources***

*Benefit of shared space to innovate*

In each network, the CST was co-located within a shared space (Network 7) or shared office building (Network 9), bringing benefits to team members and people with diabetes attending the service. Specifically, shared space facilitated “ *untimed meetings”* (Dietitian#1) to become familiar with team members and other clinicians working in different chronic condition areas. It facilitated team members to engage in shared learning, and access knowledge and information informally (e.g., discussing shared cases confidentially within shared space). The change manager echoed some of these perspectives, citing the “ *bond*” between team members that was enabled by co-location, and the “ *informal chats”* that might not be scheduled but are an opportunity to “ *bounce something off”* another clinician.

Teams were able to arrange joint (Network 7) or sequential (Network 9 and Network 7) appointments. Members of the CST perceived these appointments to be beneficial to the person with diabetes as it reduced cost (travel), improved accessibility as service delivered locally, reduced the burden on them to repeat their medical history, and allowed the team to “ *strike when the iron was hot”* (CNS#1) to address different patient needs. Clinicians in Network 7 felt that by sitting in on each other’s appointments, they benefited from the opportunity to build rapport with the patient and learn from one another, accessing discipline-specific knowledge.

*“When I [nurse] see the patient, I see a gap in need for the dietitian to give out part of the care, and may, you know, come on also a foot issue during my examination of the feet. And then to have the luxury of having a dietitian on my right hand and on my left hand a podiatrist, I feel very equipped to deliver good diabetes care.”* (CNS#1)

*Insufficient administrative staffing*

CST members in both networks flagged the lack of administrative staff as a key barrier to implementation, a concern echoed by the change manager. Administrative support was necessary to facilitate the implementation of several components of the integrated care service, enhancing team working (facilitate booking of joint appointments and diary management), supporting management of referrals and coordination of appointments/patient education (scheduling and issuing reminders).

*“The one thing that I think is lacking in the project from the word go was administrative support. … we do have some support, for just one hour a day, which is huge for us, but you know it’s still inadequate for the needs, we are three clinicians with three diaries. So that takes a lot of time and that’s very necessary work for the wheels to turn for each of us.” (CNS#1)*

***Access to information and knowledge***

*IT systems supporting and hindering team working*

Implementation was influenced by the degree to which IT systems piloted as part of the project (a) were compatible with the diabetes CST work processes, and (b) enabled information sharing and access. The presence or absence of IT systems influenced how the CST worked as a team, managed referrals, and conducted patient appointments. The challenges with IT systems manifested differently across the two networks. In Network 9 the main IT barrier was the incompleteness of the clinical database at the start of the project (e.g., only a small proportion of the dietitians’ patients were registered on the podiatrist database), and this impacted on the teams’ ability to facilitate joint appointments. In contrast, in Network 7, a shared IT system with hospital outpatient diabetes service enabled CST members to have access to one another’s notes which reduced the need to repeat patient histories and facilitated the coordination of appointments. Having a shared system that "talks to the hospital and talks to us" facilitated teamwork between hospital-based and community-based specialist team, including fortnightly multidisciplinary team MDT case discussion meetings between the CST and the consultant endocrinologists and hospital team in CHN7, which were highly valued by the team. Being able to access such data was preferable to, and more reliable than, seeking that information during patient consultations.

The lack of access to integrated IT systems across general practice, primary care and the hospital service was flagged by GPs and PNs as a barrier to providing coordinated and integrated care for people with diabetes attending both general practice and outpatient services for their diabetes care.

***Networking and communication***

*New ways of networking within the team*

Team members in both sites highlighted the quality of networking and communication, for example, *“knowing how to get hold of the person”* (CNS#2) or “ *who to ask”* (Dietician#1), and the capacity to link with team members by phone or email or in a shared space, as a key facilitator of delivering the integrated care service. Networking in this way facilitated the team to arrange joint appointments, and to feel comfortable sharing knowledge about patients, to ‘ *talk through anything’* (CNS#1) This included knowledge about how things worked in the community (e.g., best approach to refer to CNS). This was particularly beneficial for clinicians coming from the hospital setting as their colleagues could “ *fill in gaps”* (CNS#1).

*“The people you are referring to, even to just know the face of the dietitian, where the dietitian is, and then with the podiatrist, you have a face on the podiatrist, and you know the podiatrist you're referring to. And I think that makes it a lot easier. Rather than just randomly writing a referral and sending it to a general address for someone” (CNS#2)*

In Network 7, networking via a team triage meeting enabled “ *huge learning”* for team members (CNS#1) as they drew on experiences and information from one another, and it facilitated booking joint appointments. Triage was good opportunity to reflect on patient care to *“stand back from a situation, it's easier to come up with a solution”* (Podiatrist#2). Patient reflections also echoed these sentiments; those interviewed also perceived good communication between members of the CST.

*“The ladies were on the same page, (the dietitian) knew my bloods, the (DNS-integrated care) knew the diet that the dietitian was discussing with me… I had met both of them separately the last occasion and both of them had fed into each other as to what was being discussed …I had my food plan, my exercise plan, my medication plan, it all seemed to work seamlessly….” (Patient#2).*

***Compatibility of CST model with existing workflows and systems***

The incompatibility of the work processes of certain clinicians, which requires them to be “ *out and about”* (CNS#3) at general practices (CNS, CHN9) and podiatry clinics (Podiatrist, CHN7), was raised as a challenge to the team approach. In Network 7, a team approach was maintained by a “ *transparent open relationship”* (CNS#1) between team members and ‘daily’ communication, albeit remotely by phone or email on days when the podiatrist was visiting clinics.

## Implementation process

### Engagement

***Building ‘rapport’ with primary care staff***

Most practices (n=13, 63%) reported having specific education/training needs relating to diabetes care including footcare (including foot assessment), managing a high morbidity population, and diabetes updates including new medications. To engage practices and address training needs, introductory and educational Zoom meetings were held in Network 7 (16 GPs and 10 PNs), and Network 9 (31 GPs and 27 PNs). In Network 9, a nurse educational webinar (11 PNs and 2 PHNs) was also held, with some F2F education, in the form of clinic shadowing (2 PNs), and a lunchtime educational workshop (13 PHNs), held in Network 7.

In both networks, engaging key stakeholders facilitated implementation, specifically supporting the team to manage referrals. Practice nurses who took part in focus groups reported a good understanding of the CST and referral pathways. Outreach to primary care practitioners generated “rapport” and supported practitioner “ *upskilling*” (Dietitian#1). This point was echoed by PNs who felt the support and education *“little tips and information”* (PN1, FG) provided by members of the CST helped to improve practice and their diabetes knowledge *.* GPs/PNs perceived the CST as having a relative advantage over other models. For example, being able to speak to the same HCP when seeking advice compared favourably to their experiences of communicating with hospital outpatient services. Both GPs and practice nurses acknowledged the difficulties in ‘keeping up’ with diabetes treatment options and outlined how the CNS supported continuous education.

*“Often if you phone up the hospital, you could get a different person, I think knowing who the person is, helps with the continuity of care, and knowing that you can speak to a person directly is invaluable”. (GP#3)*

Engagement between the CST and primary care practitioners also aided follow-up contact and clarifications regarding referral criteria. GPs and practice nurses found it easy to access the CST service and this continuous/consistent link allowed them to have patients seen quickly when necessary or simply to ask for “ *quick word of advice”* (GP#2) from the CST when a referral was not deemed necessary. In Network 7, the team engaged with the Network GP Lead recruited as part of the new community healthcare networks, to decide how best to format the referral form to facilitate joint referrals and subsequent triage. This education/training and engagement was particularly important given clinicians at both sites received inappropriate referrals and needed to provide practices with guidance.

*“I suppose the other learning is it’s just really important to engage with the GP practices. They were very happy to have I think like designated people that they can approach if they've any queries or I suppose just upskilling practices on referral criteria and referral forms and stuff like that. It really helps them understand.”* (Podiatrist#1)

Sometimes inappropriate referrals (referrals which did not meet the referral criteria) were accepted as CST members recognized the GP may be busy, particularly due to COVID-19. For example, one CNS considered it a better use of the service to ensure the patient “ *gets educated on diabetes rather than getting fussy about the actual criteria”* (CNS#2). GPs and practice nurses saw it as an indicator of good working relationships with members of the CST that informed discussions could take place on individual patient needs for specialist services where patients might not necessarily meet ‘eligibility’ criteria on paper. However, the “ *tendency*” to accept inappropriate referrals, and the downstream impact on capacity, was flagged by the change manager as a barrier, with implications for the sustainability of the service. 

### Importance of planning implementation

CST members highlighted the importance of taking some time at start of the service to *“get the process and structure [of the service] all clear”* (Podiatrist#2) before seeing patients (e.g., triage, planning and deciding workflows, checking how other services are run, what policies they have, having an “ *early conversation”* (Dietician#1) about referral pathway within and between services.

### Support from formally appointed internal implementation leaders

Leadership from the local project team and the change manager was a facilitator in both sites, providing guidance on monitoring, how to work as a team, and “ *iron out’ issues”* (Podiatrist#2) with patient caseload, and quality monitoring (e.g., providing a ‘cheat sheet’ to assist clinicians with the collection of activity of their service). The change manager also spoke about being “ *hands on”,* driving the implementation of the project through weekly project meetings “ *to keep the momentum going on a week-to-week basi*s”. In Network 9, one clinician highlighted the need for local shared leadership, as on occasion there were conflicting opinions between different discipline-specific line managers on how the intervention should work.

## External environment and context

### Needs and resources of people with diabetes

***Continuity of care and time to “unravel” issues***

Despite the challenge of shifting to remote interactions, both GPs and practice nurses flagged the benefits of continuity of care for patients attending the CST and felt that seeing the “ *same people which is very important”* (GP#3) led to greater patient satisfaction and patient engagement, that patients “ *don’t lose interest and that is the key to keeping them coming back*” (PN#4, FG). CST team members believed that the service afforded people with diabetes time to (a) develop a better understanding of diabetes, through dedicated 1:1 education with dietitian or structured education, and (b) explain and “ *unravel exactly what’s happening for them*”(CNS#1) in the appointment, to support self-management. This was echoed by patients, with 88% (n=36) reported ‘definitely’ having enough time to discuss their diabetes care and 93% (n=37) felt they had been provided with the ‘right amount’ of information to help them manage their diabetes. Most patients perceived they were involved as much as they wanted to be in discussions about their diabetes care (78%, n=32) and ‘definitely’ feeling more confident about managing their diabetes following the consultation (73%, n=30); one female interviewee who had attended all three HCPs spoke of having a “ *collaborative interaction on each visit*” (Patient#9). Scores on the individual items on the 5-item CARE person-centred measure were high with most participants responding ‘Excellent/Very good’ to each statement.

GPs and nurses felt that general practice was well-positioned to deliver structured diabetes care in the primary care setting; overall, 87% of respondents had a diabetes register (n=13) and most (n=11, 73%) used a recall system to schedule diabetes review visits. However, they perceived members of the CST had more time for patient education and support compared to GPs, and valued access to the CST specialist support as and when needed for patients who might be *“struggling”* with their diabetes care and need a “ *steer in the right direction”* (PN#6, FG *).* The intensive support provided by the CST to encourage people with diabetes to engage in their care, whereby *“all of a sudden there's maybe three people looking out for different areas*”(Dietitian#2), was highlighted as a benefit. All patient interviewees found the service accessible (in terms of wait times and travel) although qualitative feedback on the questionnaire indicated that phone appointments were an issue for those with hearing problems.

## Characteristics of the CST

### Adaptability

***Adapting mode of care delivery due to COVID-19***

**Figure 2.**
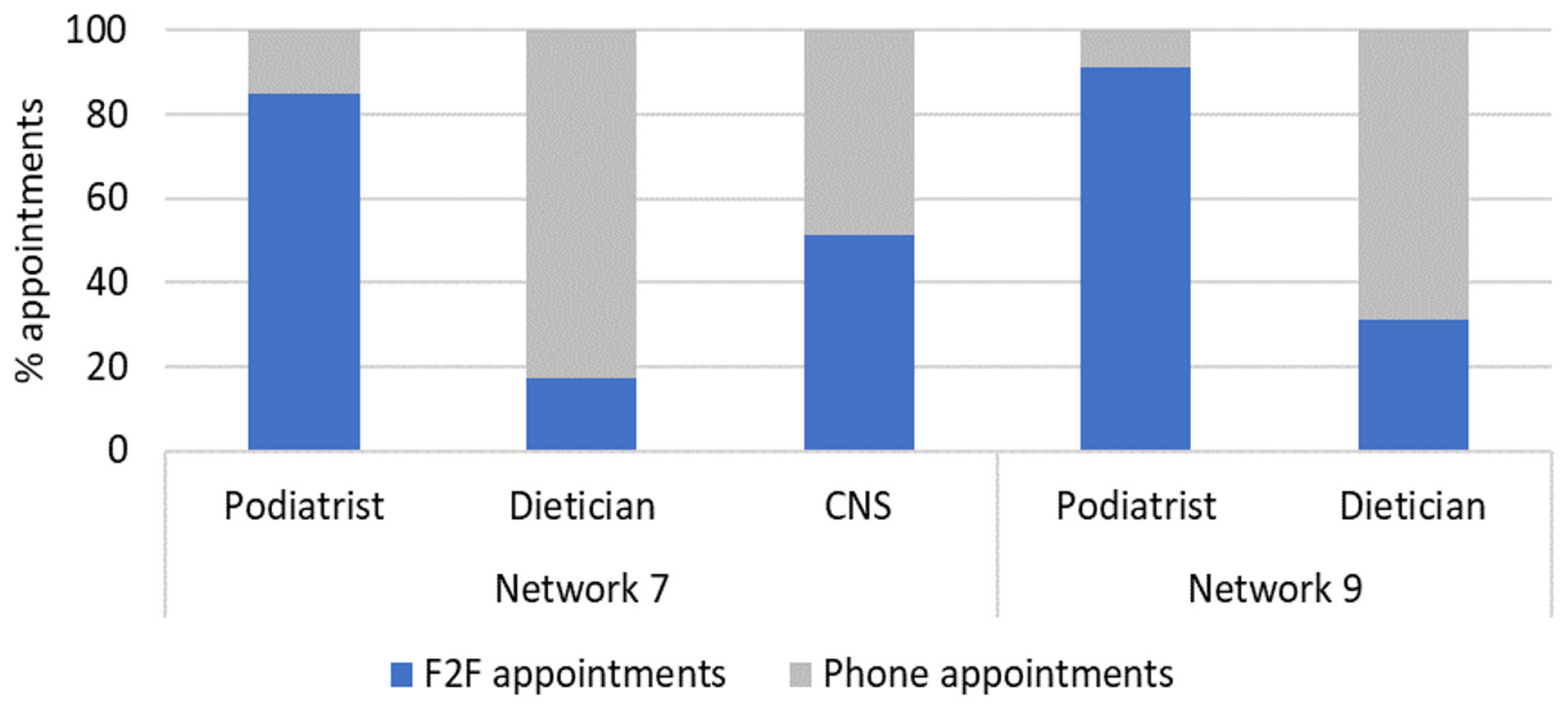
Appointment delivery mode across CST clinicians in Network 7 and Network 9*. *Note that data on appointment mode was not collected by existing CNS in Network 9.

The CST adapted in response to COVID-19, changing how different aspects of the service were delivered. The teams adapted to provide a mix of F2F and phone appointments ( Figure 2) to reach patients who might be hesitant or unable to attend appointments in person. Due to Covid-19, the delivery of professional education was limited in both networks over the project evaluation period. Despite challenges, teams adapted; as mentioned, the CST delivered networking and educational events for HCPs via Zoom.

At a general practice level, practices varied in terms of how they adapted to the COVID-19 restrictions; 50% (n=7/14) of practices reported that they had temporarily paused structured routine diabetes reviews for people with type 2 diabetes (remove/skipping elements), while the remainder reported continuing with mostly face-to-face reviews. Most practices that had stopped face to face reviews resumed them when restrictions eased [all practices in Network 7 (n=5) and 80% in Network 9 (n=8)].

## Discussion

### Summary

Few studies have examined the process of implementing linked community specialist teams as a strategy to support delivery of integrated diabetes care ^20, 22, 23^. Drawing on CFIR to integrate different stakeholder perspectives, we contribute to an understanding of the *process* of implementing a CST assigned to a designated population to support integrated care, including barriers and enablers. Specifically, in terms of the internal context we highlighted how the CST capitalised on shared space, the value of enhanced networking between team members and leadership from the change manager, and the challenges to joint working presented by lack of access to information and limited resourcing of administrative staff. With respect to implementation process, engagement to build “ *rapport”* with general practice staff, and the need to adequately prepare before implementation, clarifying processes and structures within the team prior to seeing patients were considered key. Lastly, we illustrated the adaptability of such strategies, how teams work around barriers to implementation; COVID-19 meant shifting to remote delivery of patient consultations and virtual meetings to engage health professionals.

### Implications and lessons learned

The Irish health service is undergoing major reform to address the population’s changing healthcare needs. As mentioned, the alignment of primary care services with the new community healthcare networks, forms part of the Enhanced Community Care Programme introduced 2020–2021 ^50^, which aims to enhance community health services and reduce pressure on hospital services. As part of these reforms, Ambulatory Care Hubs are being established, providing specialist services in the community, each serving approximately 3 CHNs. New CSTs for chronic disease continue to be rolled out but will be situated in the hubs. As such, the four key recommendations based on the findings of this current evaluation may be relevant for the ongoing and future delivery of CST.

***Support opportunities for informal learning and interprofessional collaboration***

Co-locating specialities is proposed as an important strategy to support service integration ^51^, promote collaboration, increase efficiency and reduce physician burnout ^52^. Research to date has shown co-location can support ‘corridor conversations’ about patients ^53^, informal learning ^54^, interactions which can support interprofessional collaboration ^55^ and a better understanding of colleagues’ roles and responsibilities ^54, 56^. In this study, sharing space with colleagues was particularly beneficial for clinicians coming from the hospital setting as their colleagues could inform them about how things worked in the community. This suggests the importance of local organisational knowledge and enabling such knowledge to be shared among clinical teams. In this study we found that shared space served as the catalyst for several elements such as: early service planning to clarify processes and structures such as triage and referral forms; relationship-building; information-sharing; and joint or sequential appointments. The latter were perceived to improve accessibility and reduce the burden on patients, substantiated by patients’ positive reflections on wait-times, involvement in discussions about their care, and confidence to manage their diabetes following engagement with the CST. Few studies have looked at the effectiveness of joint appointments for adults with diabetes. There is existing research on joint appointments (between GP, CNS and consultants) ^57, 58^ and CNS and dieticians implemented as part of a new integrated care service, including triaged referral pathway to community-based specialist team. This research suggests joint appointments led to possible improvements in glycaemic control ^57^ and reduction in the number of appointments ^58^ but more robust research is needed to establish effectiveness. In one network, joint appointments were perceived to facilitate more intensive management which aligns with research on the implementation of co-located community teams in the UK, which cited informal information sharing and case discussions as enabling more rapid action to be taken to address patient issues ^56^. 

However, there are a few important considerations with respect to the co-located CST model. First, depending on the type of patients, specialities may not share as many patients as expected. For example, a podiatry service may see more complex patients, while a dietitian service could see more newly diagnosed/early disease patients (likely to be low/moderate risk for diabetic foot disease and not attending podiatry yet). Furthermore, in the Irish health system boundaries for access to different professionals can vary, meaning an individual’s eligibility for different services may not be consistent. For example, in Network 9, referral was based on different geographical boundaries; a referral to the dietician was based on the address of *GP* in the network and a podiatry referral based on *patient* address in the network. Second, implementation of the co-located model may not be practical or compatible with existing workflows. Our study identified practical limitations of the model in scenarios where CNS visit practices and podiatrists attend clinics in the community and may not be consistently based within a shared space. Different models of co-location for diabetes care exist. These include co-location of multidisciplinary teams *within* general practice ^22, 59^, running clinics adjacent to GP and nurse rooms ^20^ and on-site consultation with a diabetes expert team (specialist physician and nurse specialist) ^60^. These are models which can offer advantages for patients in terms of being closer to home ^61^ and still provide continuity of care by a dedicated team ^59^, something valued by practice staff in both networks in the current study. Furthermore, co-location may not always be feasible (for example due to staff turnover and lack of permanency in a shared space ^56^), or appropriate. For health networks with less condensed populations, centralising co-located teams may extend travel time for some patients. Lastly, while co-location may facilitate joint or sequential appointments or more intensive responses to patient needs, it requires greater flexibility or protected time in clinician diaries to ensure feasibility. Overall, the aim should be to consider how to support coordination, networking, and communication between clinical team members regardless of physical location.

***Foster ‘rapport’ with primary care services***

Dedicated engagement opportunities and educational events with general practices were important facilitators of implementation. Having access to the CST for advice was considered key to reduce inappropriate referrals. The process of building trust and relationships between stakeholders, termed collaborative governance, has been identified by Baltaxe *et al*. as a mechanism of the implementation of integrated care, which establishes a ’shared narrative’ and understanding of the programme among stakeholders ^62^. Given the findings from the current study, having outreach and educational meetings as strategies alongside a CST, seems key to achieve this aim. Importantly, the current study illustrates that this type of engagement can be achieved remotely, which may ultimately mean outreach and education is more accessible.

***Develop the core infrastructure to underpin innovation***

Challenges to team working were exacerbated by new ICT systems and limited functionality and interoperability in established ICT systems. The need for robust IT infrastructure to support service integration is an issue that has been flagged previously in Ireland ^63^ and internationally ^12, 26, 56, 62, 64^.

Perhaps less frequently highlighted in the literature on the implementation of integrated care, is the important role of administrative staff to support specialist teams. As illustrated through this study, administrative staff are considered necessary to support service monitoring, information sharing and coordinate referrals to allow for more responsive service delivery and joint appointments and better use of clinician time. International examples on the integration of mental health and social care services ^65^, and implementation of integrated diabetes care in the Netherlands ^26^ have flagged the substantial workload posed by administrative duties which could detract from clinical care.

***Appoint internal implementation leaders***

Facilitation is often used as a strategy to address barriers and support implementation of interventions ^42, 64^. The importance of ‘hands on’ facilitation and leadership to support implementation of service changes ^67^, and integrated care ^9, 54, 64^ has previously been highlighted. In this study, facilitation was operationalised by a change manager who kept implementation on track, provided project management support, and was able to troubleshoot issues. This type of role will continue to play a part in future reforms; within the new specialist ambulatory hubs in the community, there will be Operational Leads or Team Coordinators providing local leadership. While the formal leadership represented by the change manager was important, there was also some evidence of “collective leadership” which may have contributed to successful implementation. CST had some ownership over the service in their respective networks, taking on leadership roles when planning service delivery, including workflows, triage, referral pathways and approaches to engage general practice staff.

## Strengths and limitations

This study is strengthened by integrating multiple perspectives, both professional groups and people with type 2 diabetes. However, this is a study of a CST as a strategy to support integrated care within a particular context, one which is characterised by an ongoing reform programme to formalise structured diabetes management in general practice, and address deficits in the availability of specialist support in the community. As such, the lessons learned here with respect to introducing co-located clinical specialist teams, alongside educational support and facilitation may be less relevant to health systems with a more robust community healthcare infrastructure. While we sought feedback from patients about their experiences attending the CST, we do not know the total number of people with type 2 diabetes in each network and the level or quality of care they received. Moreover, the numbers of patients who saw more than one clinician or who had joint/sequential appointments was not recorded so we cannot determine the profile of patients who may be eligible or benefit from this model. We also do not know the degree of overlap in terms of patients seen by podiatrists vs. dieticians vs. CNS. The study is further limited by the difficulty recruiting general practice staff to take part in the evaluation. Only three GPs were interviewed, and the response to the survey was relatively low at 52%. Practices with an interest in diabetes may have been more inclined to take part. Recruitment of GPs for research has often been flagged as challenging ^68– 70^ but was further compounded by COVID-19 as at the time of the evaluation, practices were involved in roll-out of the national vaccination programme. Despite challenges, two focus groups with practice nurses were carried out.

## Conclusion

We contribute to an understanding of the *process* of implementing a CST to support integrated care, illustrating how the team benefited from shared space, enhanced networking, and leadership, and worked around barriers (COVID-19) to implementation. However, implementation must be supported by a core infrastructure (IT systems and staff). The practicalities of co-location and the relative advantages of different delivery models are important considerations. Lastly, further work is needed to evaluate the clinical effectiveness of this integrated model of care.

## Ethics and consent

Ethical approval to conduct the evaluation was obtained from the Clinical Research Ethics Committees of the Galway University Hospital (C.A. 2493) and UCC Research Ethics Committee (ECM 4 (r) 13/4/2021) on November 6 ^th^ 2020 

Freely-given written informed consent to participate in the study and for the study findings to be published was obtained from all participants. Participants were given a copy of an information leaflet and consent form before data collection and asked to return a signed consent form to the research team.

## Data Availability

The underlying data are not available for this article. As this work pertains to a small evaluation of a pilot project within the health service, ethical approval was not received to share the data beyond the research team, with third parties. The following text appears in the consent forms: ‘ *Only the research team will know that you are taking part. Your data will not be shared with any third parties’;* therefore, we do not have consent to share participant data with third parties. Moreover, given the small number of participants from identifiable geographic locations (specific and named community healthcare networks), we feel it would not be possible to de-identify and share data without compromising confidentiality. This is true of both interview/focus group and survey data; variables such as practice size, staff number, and network location could make practices identifiable but removing them would compromise the usefulness of the dataset. Zenodo: Slaintecare Integration Fund Evaluation extended data: Topic Guides, questionnaires and Questionnaires (patient and health care professionals), referral criteria, and details of barriers to and facilitators of service components.). https://doi.org/10.5281/zenodo.7271951 This project contains the following extended data: Patient experiences questionnaire.pdf GP questionnaire.pdf Patient topic guide.pdf HCP topic guide.pdf GP_PM Topic Guide.pdf GP referral / eligibility criteria for the CST Barriers and facilitators of components of the integrated care service Data are available under the terms of the Creative Commons Attribution 4.0 International license (CC-BY 4.0).
