# Supplementary material for: Implementing a community specialist team to support the delivery of integrated diabetes care: experiences in Ireland during the COVID-19 pandemic
Source: HRB Open Res. 2023 Jan 5;6:1. [Version 1] doi: 10.12688/hrbopenres.13635.1 (PMC10333780; doi:10.12688/hrbopenres.13635.1)
Supplement: Peer review history

# Peer review: Reviewer response for version 1

**DOI:** 10.21956/hrbopenres.14908.r33984

**Published:** 2023-07-28

**Authors:** Cesare Miranda

**Affiliations:** 1 Pordenone Hospital, Pordenone, Italy

The COVID-19 pandemic has had a strong impact on the treatment of all diseases, especially chronic ones, and diabetes is no exception. The COVID-19 pandemic has favored the adoption of a new model of assistance delivery to facilitate the delivery of remote assistance to patients.

 Implementing integrated diabetes care is critical but not easy to achieve.

 I believe that strengths of this study are the fundamental role of the multidisciplinary team and therapeutic education in diabetes care while the limitations are the lack of efficient administrative support and the the lack of access to integrated IT systems across general practice, primary care and the hospital service.

 The current study is on a topic of relevance and general interest to the readers of the journal.

 I found that publication is interesting. The design is justifiable, the rationale is clear, the Introduction is useful. Materials and methods are well described. Data and results are clearly provided.

 Limitations of this study are pointed out by the authors. No corrections.

Is the work clearly and accurately presented and does it cite the current literature?

Yes

If applicable, is the statistical analysis and its interpretation appropriate?

Yes

Are all the source data underlying the results available to ensure full reproducibility?

Yes

Is the study design appropriate and is the work technically sound?

Yes

Are the conclusions drawn adequately supported by the results?

Yes

Are sufficient details of methods and analysis provided to allow replication by others?

Yes

Reviewer Expertise:

diabetes, diabetic foot, peripheral arterial disease, peripheral neuropathy

I confirm that I have read this submission and believe that I have an appropriate level of expertise to confirm that it is of an acceptable scientific standard.

# Peer review: Reviewer response for version 1

**DOI:** 10.21956/hrbopenres.14908.r34735

**Published:** 2023-07-10

**Authors:** Cecilia Jimeno

**Affiliations:** 1 Division of Endocrinology, Diabetes and Metabolism, Department of Medicine, Philippine General Hospital & Department of Pharmacology and Toxicology, College of Medicine, University of the Philippines, Manila, Philippines

This study aimed to evaluate the implementation of a multidisciplinary diabetes Community Specialist Team (CST) to support the delivery of integrated type 2 diabetes care during COVID-19 in two health networks in Ireland. Using a mixed-methods design this study gave an overview of implementation status, barriers and facilitators to implementation, and adaptations during the pandemic. The team had a substantial number of consultations even during the pandemic, and there was hardly any delay before being seen by a health care professional. Facilitators of success included leadership from local project teams and change managers, and shared space enhanced networking. Lack of administrative support for the CST was a barrier.

 The methodologies used in this study are well designed and elaborated, and were explained clearly. However, the manuscript even as early as the abstract had many abbreviations that were not previously given yet but which then appear e.g. CNS. While the results are also unquestionable, the major limitation which the authors also acknowledged is the low response to the survey and the limited number of GPs who were interviewed. Perhaps, given thee limitations, future studies can validate the results by including a bigger number of participants for the surveys with the GPs better represented. 

 Finally, while the source data is not given or available, the authors have at least provided a link to the tools that were used during the study such as questionnaires and topic guides, so that any future investigators can do similar studies and validate their results.

Is the work clearly and accurately presented and does it cite the current literature?

Yes

If applicable, is the statistical analysis and its interpretation appropriate?

Yes

Are all the source data underlying the results available to ensure full reproducibility?

No

Is the study design appropriate and is the work technically sound?

Yes

Are the conclusions drawn adequately supported by the results?

Yes

Are sufficient details of methods and analysis provided to allow replication by others?

Yes

Reviewer Expertise:

Diabetes, endocrinology, pharmacology

I confirm that I have read this submission and believe that I have an appropriate level of expertise to confirm that it is of an acceptable scientific standard.

# Peer review: Reviewer response for version 1

**DOI:** 10.21956/hrbopenres.14908.r33980

**Published:** 2023-07-10

**Authors:** Mohamed Hassan Elnaem

**Affiliations:** 1 Ulster University, Coleraine, Northern Ireland, UK

Thanks for the invitation to review this interesting article that describes the implementation of CAT to support integrated care delivery.

 The authors have put in nice efforts in writing this paper. However, here are my few comments for further improvements:

- The title is about implementing a service rather than evaluating it, as stated in the objectives. My suggestion is to standardise the term for describing the implementation process.
- Methods: A mixed-method approach was followed. However, the methods section was not organised clearly to present the approach followed. I suggest that the author create a main heading for each qualitative and quantitative category and then discuss relevant subheadings under each one.
  Moreover, it was not clear for the quantitative part how the survey was developed, validated, and the number of responses calculated.
- Results section: It is good to reorganise it in a consistent way with the two main study parts.
- Discussion: This section needs major rearrangements. It was not clear why the authors stated implications early in the discussion before highlighting the implementation issues that appeared later on. I believe this section will benefit from thorough revisions to the flow of presented ideas.
- Limitations: the exact involvement of each team throughout the stages was not justified. Was there any planned involvement of pharmacists as care team members? Is it relevant in the study setting to consider this point?

Thanks and regards.

Is the work clearly and accurately presented and does it cite the current literature?

Partly

If applicable, is the statistical analysis and its interpretation appropriate?

Partly

Are all the source data underlying the results available to ensure full reproducibility?

Yes

Is the study design appropriate and is the work technically sound?

Yes

Are the conclusions drawn adequately supported by the results?

Yes

Are sufficient details of methods and analysis provided to allow replication by others?

Yes

Reviewer Expertise:

Clinical pharmacy, quality use of medicine, diabetes care

I confirm that I have read this submission and believe that I have an appropriate level of expertise to confirm that it is of an acceptable scientific standard, however I have significant reservations, as outlined above.

# Peer review: Reviewer response for version 1

**DOI:** 10.21956/hrbopenres.14908.r33670

**Published:** 2023-06-22

**Authors:** Kirsty Winkley

**Affiliations:** 1 Florence Nightingale Faculty of Nursing, Midwifery and Palliative Care, King's College London, London, UK

This was an evaluation of the implementation of a community specialist diabetes team to support delivery of integrated diabetes care in Ireland. This used a mixed methods approach to implementation and evaluation of the new service. It was introduced during the COVID-19 pandemic which had an impact in terms of the nature of the service that could be delivered and the research in terms of engagement. The implementation itself was guided by an appropriate strategy, the Expert Recommendations on Implementing Change (ERIC), research framework, i.e. the Consolidated Framework for Implementation Research (CFIR) and reporting framework, Standards for Reporting Implementation Studies (StaRI). The report is detailed and the main results are synthesised and triangulated and the main barriers and facilitators of implementation are reported as well as the benefits to people with diabetes who accessed the service. The discussion is comprehensive in terms of how and what challenges remain to be addressed. The main strengths and limitations are noted. In terms of improving the current report I would suggest that the authors work on the following minor points:

1. Abbreviations, ensure that all abbreviations used are given in full before first abbreviation. There is currently some inconsistency, e.g. in the abstract some are given and some are not, but are then given in the main text.
2. Spelling, again for consistency, ‘dietician’ and ‘dietitian’ are used inter-changeably. World Health Organization (note American English spelling).
3. The methods section would benefit from some specific objectives in addition to the overall main aim/s of the project.
4. Note that a diabetes consultant provides support to the community diabetes team but they were not interviewed. Was this a potential limitation?
5. Ethics registration given at the end of the discussion, should this also be added to the methods.
6. In the methods section, the survey data includes some free text responses, how were these qualitatively analysed, i.e. content analysis?
7. Note that there was no data on DNAs, would this have been useful in terms of evaluation?
8. Not sure why there are 2 different types of qualitative analysis reported, for the CST and the other stakeholders, could the authors provide justification.

Is the work clearly and accurately presented and does it cite the current literature?

Yes

If applicable, is the statistical analysis and its interpretation appropriate?

Yes

Are all the source data underlying the results available to ensure full reproducibility?

No source data required

Is the study design appropriate and is the work technically sound?

Yes

Are the conclusions drawn adequately supported by the results?

Yes

Are sufficient details of methods and analysis provided to allow replication by others?

Yes

Reviewer Expertise:

type 2 diabetes, psychological aspects of diabetes care, nursing

I confirm that I have read this submission and believe that I have an appropriate level of expertise to confirm that it is of an acceptable scientific standard.
